# Neonatal Diesel Exhaust Particulate Exposure Does Not Predispose Mice to Adult Cardiac Hypertrophy or Heart Failure

**DOI:** 10.3390/ijerph13121178

**Published:** 2016-11-24

**Authors:** Yonggang Liu, Chad S. Weldy, Michael T. Chin

**Affiliations:** 1 Division of Cardiology, Department of Medicine, University of Washington, Seattle, WA 98109, USA; ygliu99@u.washington.edu (Y.L.); chad.weldy@duke.edu (C.S.W.); 2 Center for Cardiovascular Biology, School of Medicine, University of Washington, Box 358050, 850 Republican Street, Room 353, Seattle, WA 98109, USA

**Keywords:** diesel exhaust particulates, cardiac hypertrophy, heart failure, air pollution, PM_2.5_

## Abstract

*Background:* We have previously reported that in utero and early life exposure to diesel exhaust particulates predisposes mice to adult heart failure, and that in utero exposure alone is sufficient to confer this predisposition. This follow up study addresses whether neonatal exposure alone can also confer this predisposition. *Methods:* Newborn male C57BL/6 mice were exposed to diesel exhaust (DE) particulates immediately after birth until weaning at 21 days of age, whereupon they were transferred to filtered air (FA) conditions. At the age of 12 weeks, transverse aortic constriction (TAC) was performed followed by weekly echocardiography for three weeks. After the last echocardiogram, mice were euthanized for organ harvest, gravimetry and histology. *Results:* Neonatal exposure to DE particulates did not increase susceptibility to cardiac hypertrophy or heart failure after TAC when compared to FA exposed controls (ventricular weight/body weight ratio 7.505 vs. 7.517 mg/g, *p* = Not Significant (NS)). The left ventricular ejection fraction after TAC was similar between groups at one week, two weeks, and three weeks after procedure. Histological analysis showed no difference in the degree of cardiac hypertrophy or fibrosis. *Conclusions:* Neonatal exposure to DE particulates does not predispose mice to TAC-induced cardiac hypertrophy and heart failure in adulthood, in contrast to previously published results showing susceptibility due to in utero exposure.

## 1. Background

Epidemiological studies conducted over the last two decades have demonstrated a strong association between exposure to air pollution and cardiovascular disease (reviewed in [1,2,3,4,5]). Although the predominant cardiovascular complication of chronic exposure to air pollution is ischemic heart disease, statistically significant associations are also seen for arrhythmias, heart failure and cardiac arrest [6]. Although adult exposure to the fine particulate matter component of air pollution (PM_2.5_) has been specifically associated with exacerbation of mortality [6,7,8], the potential cardiovascular effects of in utero and early life exposure are largely unknown. In one study, in utero exposure to diesel exhaust (DE) led to gender related differences in obesity in the offspring [9]. In human populations, prenatal exposure to diesel particulates is associated with a decrease in placental mitochondrial DNA content [10], an increased incidence of preeclampsia [11] and a small but measurable decrease in birth weight [12], while postnatal exposure affects lung development [13,14,15]. We have previously reported that maternal DE exposure for three weeks prior to timed mating, with continued exposure during gestation and postnatally through either weaning or to 12 weeks of age, predisposes mice to adult heart failure after transverse aortic constriction (TAC) and also alters pulmonary cytokine expression when compared to filtered air (FA) exposure [16]. We have also found that in utero exposure alone is sufficient to confer adult susceptibility to heart failure, through a mechanism that involves placental injury, inflammation and vascular oxidative stress [17]. This study addresses whether neonatal exposure alone is sufficient to confer adult susceptibility to cardiac hypertrophy and ventricular dysfunction in a TAC model.

## 2. Methods

### 2.1. Animals

Wild type male C57BL/6J mice (12 weeks old) were used for these studies. Mice were obtained from Jackson Laboratories (Bar Harbor, ME, USA). The studies were conducted according to guidelines and protocols approved by the University of Washington Institutional Animal Care and Use Committee. The protocol number is 4134-01 and the date of approval is 13 June 2016.

### 2.2. Transverse Aortic Banding

Mice underwent transverse aortic banding surgery or sham surgery as previously described [16,17,18,19,20]. Transverse aortic banding was performed using a 27 gauge needle. Echocardiography was performed every week after the surgery until 3 weeks after the surgery.

### 2.3. Diesel Exhaust Exposure

**Figure 1:**
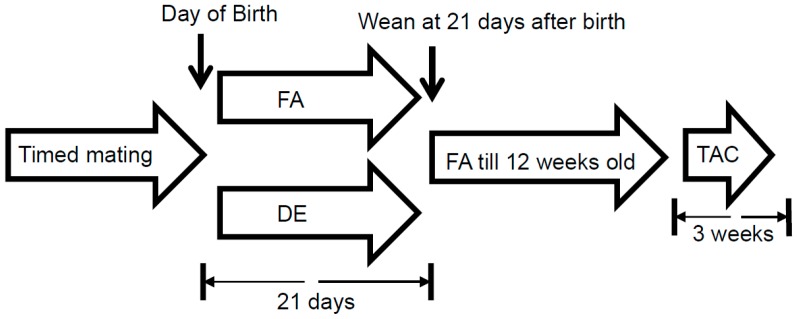
Schematic diagram of the neonatal diesel exhaust particulate exposure protocol. FA: filtered air; DE: diesel exhaust; and TAC: transverse aortic constriction.

Wild type male and female C57BL/6J mice were transferred to our diesel exposure facility where exposures to either FA or DE were conducted simultaneously in an Allentown caging system with positive/negative ventilation (Allentown, NJ, USA) under Specific Pathogen Free (SPF) conditions. After brief recovery from transfer, the mice underwent timed mating. Newborn pups were exposed with their dams from birth (P0) to either FA or DE until weaned at 21 days (P21) (Figure 1). DE (300 µg/m^3^) was generated from a single cylinder Yanmar diesel engine (model YDG5500EV-6EI) and has been characterized in terms of chemical composition as previously described [16,17,18]. Exposures were 6 h/day, 5 days/week, producing roughly a 50 µg/m^3^ cumulative time weighted average exposure. Mice were exposed in their cages containing food, bedding and water, with routine changes according to the vivarium schedule. Six litters were exposed to FA and ten litters were exposed to DE.

### 2.4. Echocardiography

Echocardiographic experiments were performed to measure heart function and chamber dimensions such as left ventricular wall thickness (LVWT), left ventricular end diastolic dimension (LVEDD), and left ventricular ejection fraction (LVEF, in %) using a VisualSonics VEVO 2100 system (VisualSonics, Toronto, ON, Canada) equipped with an MS550s scan head, as previously described [16,17,18,19,20]. Mice were lightly anesthetized with 1% isoflurane when performing echocardiography. Data were accumulated in M mode from the short axis and LVEF (in %) was calculated as previously described [16,17,18,19,20].

### 2.5. Gravimetric Analysis and Histopathology

Gravimetric analysis was performed as previously described [16,17,18,19,20]. The mice were euthanized by carbon dioxide inhalation followed by weighing, heart removal and exsanguination. Atria were removed and ventricular weight changes between groups were tabulated as mg ventricular weight (VW)/g body weight (BW). Masson trichrome staining was used to delineate the fibrotic tissue in both perivascular and interstitial regions, and quantification of fibrosis was performed as previously described [16,17,18,19,20].

### 2.6. Statistical Analyses

All data were reported as mean ± SEM. The comparison between the groups was made by a Mann-Whitney test with Bonferroni correction [18]. Since we generally made 4 pairwise comparisons for each measurement, a value of *p* > 0.0125 was considered as no difference between any two groups. A value of *p* < 0.0125 was considered as significantly different between groups. All the analyses were performed using commercially available software (StatView, SAS Institute, Inc., Cary, NC, USA).

## 3. Results

### 3.1. Neonatal Exposure to DE Particulates Does Not Predispose Adult Mice to Cardiac Hypertrophy and Heart Failure

**Figure 2:**
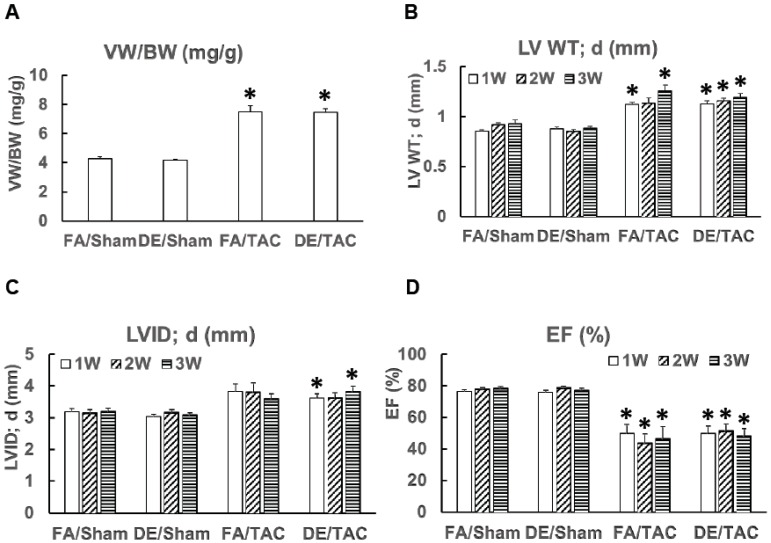
Effects of neonatal diesel particulate exposure and acute pressure overload on cardiac hypertrophy and ventricular function. Neonatal mice were exposed to DE (300 µg/m^3^) or FA (6 h/day, 5 days/week) from birth until weaning at 21 days and were then maintained in FA. Transverse aortic constriction (27 gauge needle) was done at 12 weeks of age, with serial echocardiography for three weeks post surgery, followed by euthanasia and organ harvest. (**A**) assessment of cardiac hypertrophy by ventricular weight to body weight ratio (VW/BW); (**B**) left ventricular wall thickness (LVWT) measured by echocardiography; (**C**) left ventricular internal dimension at end of diastole (LVID) measured by echocardiography; (**D**) left ventricular ejection fraction (EF) measured by echocardiography. FA/sham: *n* = 7; FA/TAC: *n* = 7 (*n* = 8 in one week and two weeks echocardiography data; one mouse died two weeks after TAC); DE/sham: *n* = 12; DE/TAC: *n* = 12; * *p* < 0.0125 compared to respective sham group. There is no statistical difference between FA/TAC and DE/TAC.

We exposed neonatal mice to DE particulates as detailed as Figure 1. Exposures were discontinued at the time of weaning and mice were housed in filtered air until the age of 12 weeks. Mice then underwent TAC at the age of three months. Heart function was measured by echocardiography for three consecutive weeks. Mice exposed neonatally to DE particulates did not show any differences in heart function when compared to FA exposed controls (Figure 2D). The left ventricular wall thickness and left ventricle cavity size changes induced by TAC were also not significantly different between the DE particulate and FA exposed groups.

Mice were euthanized three weeks after TAC surgery for gravimetric analysis. The ventricular weight to body weight ratio in mice exposed neonatally to DE particulates was comparable to the ratio in mice exposed neonatally to FA (7.505 vs. 7.517 mg/g, DE/TAC vs. FA/TAC, *p* = NS). Neonatal exposure to DE particulates thus had no effect on adult susceptibility to cardiac hypertrophy or ventricular dysfunction.

### 3.2. Neonatal Exposure to DE Particulates Does Not Increase the Degree of Cardiac Fibrosis after Pressure Overload

**Figure 3:**
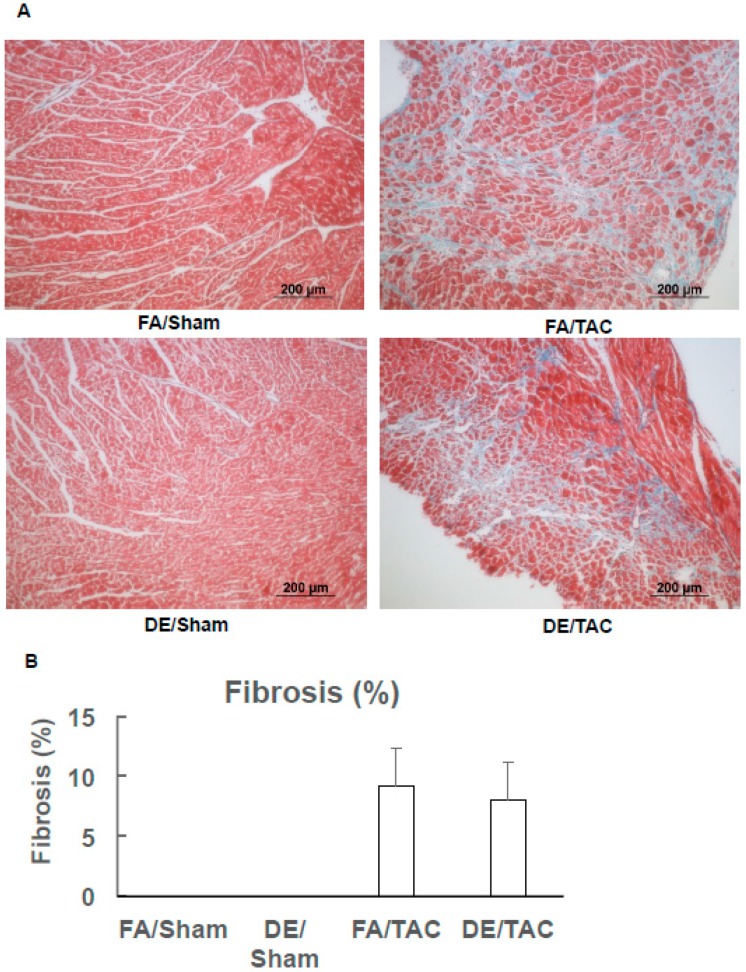
Effects of neonatal diesel particulate exposure and acute pressure overload on cardiac fibrosis. Neonatal mice were exposed to DE (300 µg/m^3^) or FA (6 h/day, 5 days/week) from birth until weaning at 21 days and were then maintained in FA. Transverse aortic constriction (27 gauge needle) was done at 12 weeks of age. Animals were euthanized for organ harvest three weeks after surgery. (**A**) cardiac fibrosis was detected by Masson Trichrome staining; (**B**) quantification of fibrotic areas as a percentage of total area by Image J software (National Institutes of Health, Bethesda, MD, USA); FA/sham: *n* = 7; FA/TAC: *n* = 7; DE/sham: *n* = 6; DE/TAC: *n* = 7. There is no statistical difference between FA/TAC and DE/TAC.

TAC induces apoptosis of cardiac myocytes and promotes fibrosis within the heart. We have previously reported that in utero and early life exposure to DE particulates leads to increased fibrosis after transverse aortic constriction [16]. We have also reported that in utero DE particulate exposure is also sufficient to promote cardiac fibrosis after TAC [17]. To determine whether neonatal exposure to DE particulates is sufficient to promote cardiac fibrosis in adult mice after transverse aortic constriction, we performed Masson trichrome staining on sections of explanted heart tissue from these mice and quantified the degree of fibrosis. One group has published a study showing that TAC surgery results in approximately 10% collagen in the mid LV at seven days and 5% at 28 days, compared to 1.5% collagen in the sham operated animals [21]. Our study shows that TAC results in 8%–9% fibrosis at three weeks after TAC, but no detectable fibrosis in sham operated animals. These differences likely result from differences in staining methodology and timing. As shown in Figure 3, neonatal DE exposure does not confer an increase in cardiac fibrosis when compared with FA exposure.

## 4. Discussion

We have previously reported that exposure to DE particulates in utero and early in life or in utero exposure alone are each sufficient to predispose to adult heart failure [16,17]. Here, we report that exposure in the neonatal period is insufficient to predispose to adult heart failure. One caveat with our study is that we terminated the study at three weeks after TAC and did not observe frank heart failure (defined as LV ejection fraction less than 30%), although we did see a significant fall in ejection fraction. We chose three weeks as our endpoint because our two previous studies showed a significant effect of gestational and early life exposure to DE on LV ejection fraction within this time frame [16,17]. It is possible that if we extended the study beyond three weeks, we may see an effect of DE, but we do not believe that this is likely. In any event, the current study contrasts with our earlier work in that no effect of neonatal exposure to DE is seen on the susceptibility to TAC-induced ventricular dysfunction. In the previous studies, gestational exposure, gestational exposure through weaning and gestational exposure through adulthood all resulted in predisposure to TAC-induced ventricular dysfunction, while exposure from P21 through 12 weeks or from 8 to 20 weeks did not confer susceptibility [16,17,18]. The most likely explanation for this discrepancy is that the window of susceptibility to DE-associated, TAC-induced ventricular dysfunction is during gestation, not the postnatal period. Other possible explanations include differences in minute ventilation in neonates compared to adult animals, the relationship of the animals’ noses to the cage opening (adults are higher) and the potential protective effects of bedding to adsorb particulates that may limit neonatal exposure in comparison to in utero exposure, although we believe that these explanations are less likely. Our future work will focus on identifying the key gestational exposure window for this predisposition, as it will be necessary for understanding and exploring the biological mechanisms that underlie this predisposition.

A variety of mechanisms have been proposed to explain the effects of particulate matter air pollution on the cardiovascular system, including direct effects of particle translocation from the lungs, induction of systemic inflammation with circulation of inflammatory mediators and altered autonomic function (reviewed in [3,5,22]). Our current finding that neonatal exposure alone is insufficient to predispose to adult heart failure, in conjunction with our previous work that in utero exposure alone is sufficient [17], demonstrates that prenatal events subsequent to maternal diesel exhaust particulate exposure determine adult susceptibility to heart failure. Although placental inflammation and circulatory defects have been observed [17], the soluble mediators and the molecular events that mediate the increase in susceptibility remain to be elucidated. Future work on identifying transcriptomic, epigenomic, signaling and metabolic effects in the exposed hearts will be essential in elucidating these mechanisms.

## 5. Conclusions

Neonatal exposure to diesel exhaust particulates does not predispose mice to cardiac hypertrophy, ventricular dysfunction or cardiac fibrosis in adulthood.
